# Refreshing the paediatric emergency medicine research priorities across the UK and Ireland

**DOI:** 10.1136/emermed-2025-215836

**Published:** 2026-06-01

**Authors:** Charlotte Sloane, Thomas Waterfield, Jordan Evans, Michael Barrett, Mark D Lyttle, Niall Mullen, performed on behalf of PERUKI, Alan Charters

**Affiliations:** 1 Wellcome-Wolfson Institute for Experimental Medicine, Queen’s University Belfast Wellcome-Wolfson Institute for Experimental Medicine, Belfast, UK; 2 Paediatric Emergency Department, Royal Belfast Hospital for Sick Children, Belfast, UK; 3 Cardiff and Vale University Health Board, Cardiff, UK; 4 Health and Care Research Wales, Cardiff, UK; 5 Emergency Medicine, Children’s Health Ireland at Crumlin, Dublin, Ireland; 6 Women’s and Children’s Health, University College Dublin, Dublin, Ireland; 7 Emergency Department, Bristol Royal Hospital for Children, Bristol, UK; 8 Paediatric Emergency Department, South Tyneside and Sunderland NHS Foundation Trust, Sunderland, UK

**Keywords:** pediatric emergency medicine, pediatric emergency medicine, research design, Patient-Centered Care, pediatrics

## Abstract

**Background:**

The original research priorities for paediatric emergency medicine (PEM) in the UK and Ireland were published in 2015. This list, generated through a modified Delphi process involving healthcare professionals only, has driven the research agenda for over ten years. With many now successfully addressed and a significantly altered healthcare landscape, there was a pressing need to refresh PEM research priorities with the input of patients and carers.

**Methods:**

James Lind Alliance (JLA) methodology was employed. The scope included unscheduled emergency care provided to children and young people irrespective of setting. An independent JLA facilitator chaired monthly steering group meetings with equal input from parents and healthcare professionals. Two online surveys were employed to generate a list of evidence uncertainties and then to prioritise research questions from key stakeholder groups (patients, carers and healthcare professionals). An online workshop subsequently used an adapted nominal group technique to reach a consensus on the top list of research priorities.

**Results:**

655 questions were submitted in Survey 1 by 338 respondents (35% patients and carers and 65% healthcare professionals). After merging questions by topic and removing out-of-scope questions, 70 summary questions proceeded to evidence reviews; three were found to have been sufficiently answered. Further merging of summary questions resulted in 46 indicative research questions for Survey 2, which received 542 complete responses (26.6% patients and carers, 73.4% healthcare professionals). The 18 highest-ranking questions were brought to the consensus workshop, in which 12 patients/carers and 15 healthcare professionals reached consensus on the 10 highest priority research questions.

**Conclusion:**

This refreshed PEM prioritisation study has identified the top 10 research priorities reflecting the views of patients, carers and a range of health professionals across the UK and Ireland. These priorities will be used to drive the PEM research agenda for the next decade.

**WHAT IS ALREADY KNOWN ON THIS TOPIC**

- Paediatric Emergency Research UK and Ireland (PERUKI) published the first paediatric emergency medicine (PEM) research priorities within the UK and Ireland in 2015, following a two-stage modified Delphi online survey of their healthcare professional membership. This list formed the foundation of PERUKI’s research agenda over the last decade.

**WHAT THIS STUDY ADDS**

- This study provides a refreshed list of the research priorities for PEM in the UK and Ireland, using the well-established JLA methodology, which is inclusive of key stakeholders’ (parents, children and young people) views throughout.

**HOW THIS STUDY MIGHT AFFECT RESEARCH, PRACTICE OR POLICY**

- This refreshed list of PEM research priorities, generated over 10 months from December 2023 to September 2024, will be used by PERUKI to direct the UK and Ireland’s research agenda for at least the next 5 years. It will be pivotal to securing grant funding for future research projects that matter most to all stakeholders.

## Introduction

 Research priority setting partnerships bring patients, carers and healthcare professionals together to ensure that healthcare research directly addresses the most important issues for all stakeholders. Within paediatric emergency medicine (PEM) the practice of research prioritisation has been established globally since 1999.^1^ In the UK and Ireland, this work has been led by Paediatric Emergency Research UK and Ireland (PERUKI),^2^ a research collaborative established in 2012 which aims to improve the care of sick and injured children through robust collaborative multi-centre research.^2^

In 2015, PERUKI published their first list of PEM research priorities,^3^ which underpinned PERUKI’s research agenda over the last decade. Using a two-stage modified Delphi process, 60 questions were gathered from their membership using online surveys. Limitations of this study included the lack of representation from patients and families, and potential for bias from healthcare professionals involved who may have had their own research agendas. Most of the initial priorities have now been addressed via multi-centre research studies, which have been instrumental in updating national guidelines and clinical practice across the UK and Ireland.^4–6^

The James Lind Alliance (JLA)^7^ is a non-profit organisation funded by the National Institute of Health and Social Care Research (NIHR). The JLA have facilitated over two hundred^8^ priority setting partnerships, using established methods to ensure the process is fair, accessible and equally represents the views of patients/carers and healthcare professionals.^7^ Research priorities identified through established methodologies such as those of the JLA are likely to influence research funders due to their rigorous and credible processes.

The aim of this Priority Setting Partnership (PSP) was to refresh the PEM research priorities for the UK and Ireland, by identifying unanswered research questions from patient, carer and healthcare professional perspectives, and to generate a final ranked list of questions.

## Methods

### Patient and public involvement (PPI)

The PSP was conducted in accordance with published JLA Methodology.^7^ A steering group of 20 members (four parents, six nurses, eight doctors, one paramedic and one academic researcher) was recruited via advertisements through online forums, PERUKI membership emails, direct contact with patient groups and social media platforms. Parents and carers received NIHR guided remuneration.^9^ Targeted involvement of children and young people within the steering group was attempted but prohibited by meeting times during school/college hours due to the timeline available for this PSP.

Selection to the steering group was overseen by a JLA facilitator to ensure fairness; only one member had previous experience of PSP participation. A list of steering group members and affiliations is on the JLA website.^10^ To ensure young people were adequately represented, a young person’s advisory group (YPAG) was consulted throughout. The PSP coordinator and lead joined online evening meetings at which the YPAG would review elements including promotional materials, questions and themes.

### Partnered organisations

Partnered organisations were selected by members of the steering group in order to engage as wide a range of respondents as possible. These included the royal colleges and associations for paediatrics and emergency medicine, paediatric research networks and relevant patient groups. As PEM treats patients with any condition, it was agreed not to approach disease specific organisations and charities in order to avoid overrepresentation for one or more conditions.

### Protocol development

The scope was inclusive of all unscheduled emergency care provided to children and young people across the UK and Ireland, irrespective of setting.^11^ Care related to adults, primary care, 111 services and inpatient care were out-of-scope.^11^

### Priority setting process

The JLA use a seven-step process to establish research priorities; creating a steering group, gathering evidence uncertainties, summarising the responses gathered, evidence checking, interim priority setting, a workshop and finally publication and promotion of results. Their process was developed in response to the limitations of traditional Delphi^12^ and expert-only methods, aiming for equitable participation between patients, carers and clinicians.

### Survey 1

The first online survey was co-designed by the steering group and YPAG, and conducted via REDCap^13^ with ‘easy-read’ and ‘advanced-read’ versions and text-to-speech functionality to increase accessibility. A hyperlink to Google Translate was signposted on both surveys to encourage participation from those who do not read or speak English. The survey was piloted with 25 children and young people, five parents, two adults with PEM experience as patients, four nurses and five doctors.

**Figure 1:**
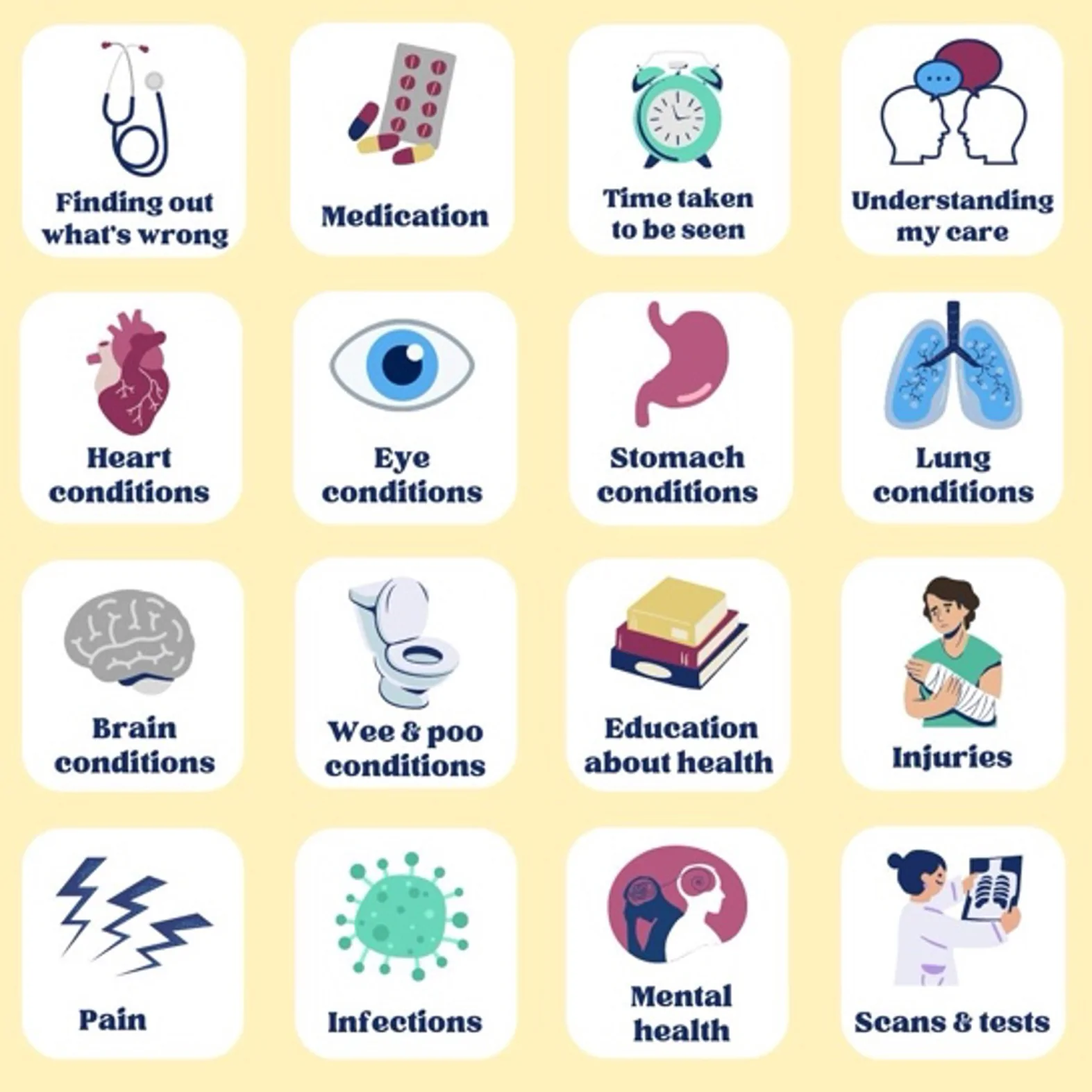
Easy read survey question prompt.

Survey 1 asked a single question: ‘What research should we focus on in children’s emergency care?’, with general topic prompts adapted from the 2015 PERUKI prioritisation exercise^3^ (figure 1 and table 1). Respondents could submit up to five free-text questions or statements that they deemed important. Demographic information was collected to ensure fair representation from all countries, ages, gender, ethnic groups and healthcare professional roles (table 2). Respondents were asked if they wanted further engagement in the PEM PSP, and if so, their details were collected; only the information specialist had access to this confidential information.

**Table 1:** Advanced read survey question prompt

Examples of topics	
Allergies	Kidneys and waterworks
Brain conditions	Life-threatening illness and injuries
Broken bones	Lung conditions
Diabetes and hormone conditions	Medications and toxins
Disease frequency	Mental health
Ear, nose and throat conditions	Menstruation (periods) and reproductive health
Education about health	Patient safety
Eye conditions	Protecting children from harm
Heart conditions	Reducing and managing pain or distress
Improving communication	Stomach conditions
Improving how healthcare is delivered	Transfer of care to adult services
Infections	Trauma
Injuries	Understanding your care
Illness prevention	X-rays and imaging

**Table 2:** Survey respondent demographic characteristics

Characteristic	Survey 1 (n=305)	Survey 2 (n=542)
Gender identity		
Female	187 (61.3)	348 (64.2)
Male	94 (30.8)	173 (32)
Female (Under 18 years old)	8 (2.6)	5 (0.9)
Male (Under 18 years old)	7 (2.3)	5 (0.9)
Non-binary	2 (0.7)	4 (0.7)
Prefer not to answer	6 (2)	6 (1.1)
Prefer to self-describe	1 (0.3)	1 (0.2)
Category of respondent		
Child or young person	16 (5.2)	20 (3.7)
Median age (range)	12 (8-18)	16 (under 7–24)
Parent or carer	69 (22.6)	110 (20.3)
Adult with PEM experience	6 (2)	14 (2.6)
Healthcare professional	214 (70.2)	398 (73.4)
Country		
England	180 (59)	342 (63.1)
Ireland	34 (11.1)	46 (8.5)
Northern Ireland	34 (11.1)	71 (13.1)
Scotland	25 (8.2)	52 (9.6)
Wales	32 (10.5)	31 (5.7)
Ethnic group		
Asian/Asian British/Asian Scottish/Asian Irish	14 (4.6)	32 (5.9)
Black/African/Caribbean/Black British	6 (2)	12 (2.2)
Mixed/multiple ethnic groups	9 (3)	10 (1.8)
White	269 (88.2)	464 (85.6)
Other ethnic group	4 (1.3)	9 (1.7)
Unknown	1 (0.3)	0 (0)
Prefer not to answer	2 (0.7)	15 (2.8)

Demographic characteristics of respondents for completed online surveys. Not inclusive of pilot survey responses. Data are presented as number (%) or median (range).

PEM, paediatric emergency medicine.

The survey was promoted on the PERUKI and JLA websites,^10^ emailed to all PERUKI members and posters displayed at PERUKI sites; paper versions were also available. The survey was also shared with partnered organisations, medical education websites, UK paramedic trusts and networks, charities, schools, online PPI groups and via social media platforms listed within the PEM PSP Protocol.^11^ Survey 1 remained open for 8 weeks (05/02/2024–31/03/2024) to maximise responses from each stakeholder group (children, young people, parents and carers, adults with experience of PEM care and healthcare professionals with experience providing PEM care).

The demographic information collected was regularly monitored, and this allowed for purposeful sampling of under-represented groups. This included encouraging minority ethnic groups to participate by highlighting this need at conferences. It also included finding ways to encourage children and young people to participate, with social media posts, contacting charitable organisations and youth groups.

### Data processing and question refinement

Complete responses were aggregated in Excel, and data were coded (with respondent IDs and question numbers) aligned with broad topics. Duplicate or similar questions were grouped and summary questions were created in lay terminology and where possible a PICO format. A JLA facilitator and independent information specialist supervised this process.

Out-of-scope submissions were coded as one of: information, policy and practice, too broad or no question asked. Examples in these categories included ‘explanation of categories and wait times’ and ‘Why can the UK not agree on one system for electronic medical records?’. Submissions were removed only after agreement by a majority of steering group stakeholders (online supplemental Data).

### Evidence reviews

**Figure 2:**
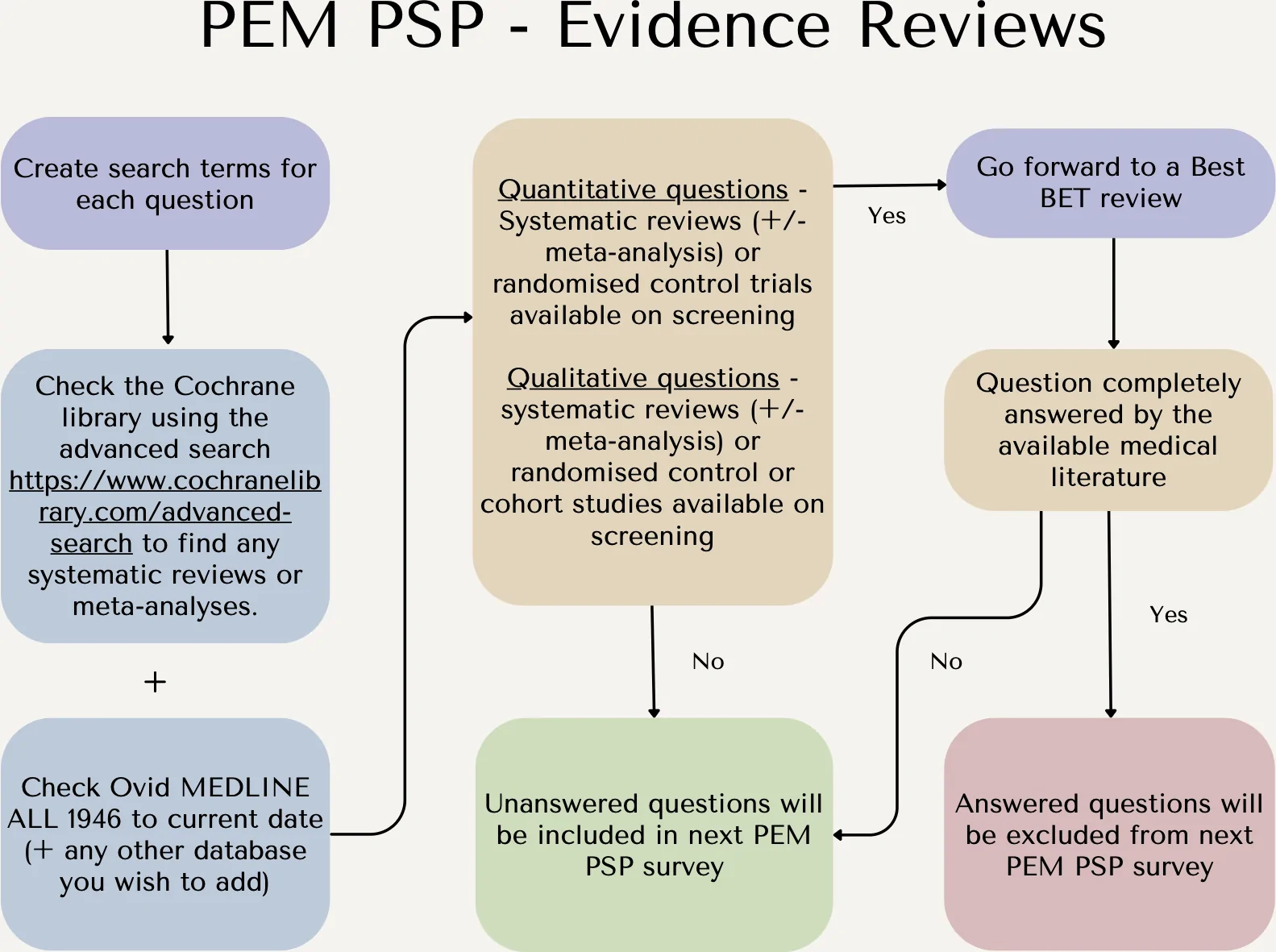
Evidence review guide. PEM, paediatric emergency medicine; PSP, Priority Setting Partnership.

The steering group produced a list of 70 summary questions for evidence review and defined the threshold for qualifying evidence including only systematic reviews and randomised controlled trials for quantitative studies. Individual literature searches for all summary questions were performed by evidence review team members (consultants, nursing consultants, resident doctors and medical students) using a guide created by the information specialist (figure 2). All questions underwent final review by the information specialist to ensure consistency. Priorities from the 2015 PERUKI exercise were reviewed and included if insufficiently answered.

Unanswered and partially answered questions were then refined by the steering group for clarity. Input was sought from the YPAG to ensure the questions could be easily understood, as further alterations to wording beyond this stage are prohibited by JLA rules.^7^ 46 questions proceeded to Survey 2.

### Survey 2: interim prioritisation

The second online survey was designed using the same approach to accessibility and remained open for 8 weeks (01/07/2024–24/08/2024). Respondents were asked for demographic information to ensure fair representation and asked to select the ten questions they felt were the most important to address within children’s emergency care.

The JLA advised a maximum of 18 questions could proceed to the workshop. Individual groups of children and young people, adults with experience of PEM care, parents or carers with experience of PEM care and healthcare professionals providing PEM care were all analysed separately to identify the top questions within each group. To ensure equitable representation, data were categorised into questions from healthcare professionals and those with lived experience of PEM care. Total scores for each group were recorded and ranked separately, then an overall average from both groups was calculated to produce a final list of priorities.

### Workshop: final prioritisation

Four JLA facilitators led the online workshop held over two half-day sessions (10/09/2024 and 11/09/2024). Participants were asked to rank the 18 questions 1 week before the workshop.

On day 1, participants were split into four breakout rooms, with an equitable mix of backgrounds. All participants were asked to talk through their ranking of questions and their reasoning, which was followed by a group discussion. Based on this, the JLA facilitator for each room adjusted each question’s rank on a shared screen and ensured the group agreed with their new rank. At the end of the session, numerical rankings were placed on the order of questions for each group and combined by the JLA facilitators.

Participants were rearranged on day two to encourage sharing of experiences and further discussion within groups. Combined rankings from day 1 were presented, with opportunity given for participants to discuss questions further. Attention was focused on questions being in or out of the top ten, rather than specifically on ranking. At the end of the second day, the rankings from all four groups were once again combined to reach consensus.

## Results

**Figure 3:**
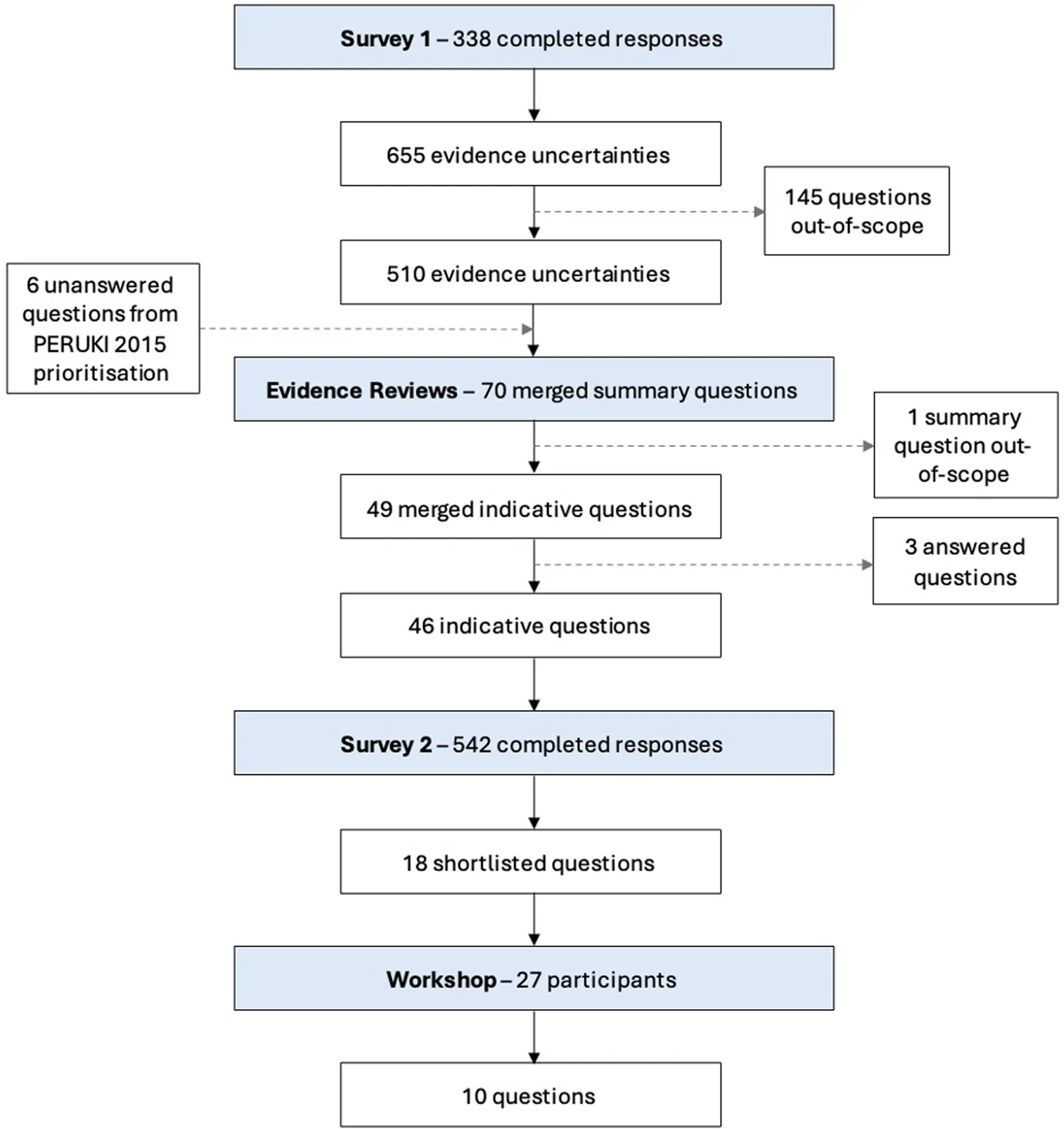
PEM PSP summary flowchart. PEM, paediatric emergency medicine; PERUKI, Paediatric Emergency Research UK and Ireland; PSP, Priority Setting Partnership.

712 respondents commenced Survey 1, with 305 completed responses and 600 questions generated. The easy read survey was completed by 63 (20.7%) respondents, and advanced read completed by 242 (79.3%) respondents. 33 respondents from the pilot survey generated 55 questions, which were added (see figure 3 for the summary flowchart). This resulted in 65% (219/338) responses from healthcare professionals and 35% (119/338) responses from people with lived experience of PEM. The demographic characteristics of the respondents are outlined in table 2.

22% (145/655) of responses were excluded as out-of-scope. 70 summary questions remained after data processing and question refinement. These underwent evidence reviews and evaluation by the steering group. Three questions were excluded at this stage, as they had been answered within the available literature. Questions underwent further refinement and merging by the steering group, resulting in 46 questions which were brought forward to Survey 2.

1021 people commenced Survey 2, with 542 completed responses. The easy read survey was completed by 109 (20.1%) respondents, and advanced read completed by 433 (79.9%) respondents. Overall, 26.6% (144/542) of respondents were people with lived experience of PEM (which fell within our target of 25%), and 73.4% (398/542) were healthcare professionals. 18 questions were shortlisted for the final workshop.

30 workshop participants were recruited from those who wished to be contacted further in Surveys 1 and 2. Following last-minute withdrawals, the workshops were conducted with 27 participants (15 healthcare professionals and 12 parents and carers). Only one member of the steering group participated to minimise potential bias. Using modified nominal group technique, a consensus of the top ten PEM research priorities was reached, see table 3.

**Table 3:** PEM PSP top 10 research priorities

1	What are the most effective ways to identify babies and children (from birth to late childhood) who are at risk of serious infection (sepsis) in emergency care?
2	What are the best ways to assess, manage, support and safely discharge children and young people with mental health conditions in emergency care?
3	What are the effects of overcrowding in emergency care on children and young people’s safety, and what can be done to improve this?
4	How can emergency healthcare professionals improve the treatment and care of serious injuries in children and young people? (trauma, eg, bleeding, head injury and chest trauma)
5	What are the best ways to assess, treat and safely discharge children (including pre-schoolers) with acute wheeze/asthma in emergency care?
6	What are the best ways to regularly monitor children in emergency care settings and ensure abnormal findings are reviewed and actioned?
7	How can emergency healthcare professionals improve communication with children, young people and their families during their care and discharge? (eg, in a way that is sensitive, age appropriate, respects their views and involves them in decision making)
8	How can emergency healthcare professionals improve how they care for children and young people with special educational needs and disabilities* (SEND)?
9	What are the best ways of assessing and sorting (triaging) children and young people who need emergency care?
10	How can emergency healthcare professionals better communicate and work with other services (eg, general practitioners, pharmacy, social care, etc) to help children, young people and families manage their conditions at home or identify the best place to go for their care if needed?

* Neurodiversity was agreed by the steering group to fall within SEND.

PEM, paediatric emergency medicine; PSP, Priority Setting Partnership.

## Discussion

This study set out to answer what are the most important research priorities within PEM to patients, carers and healthcare professionals across the UK and Ireland.

A finalised list of 18 ranked priorities from the workshop has been generated, with particular focus on the top 10. The list is transparent in its shared formation, as it is clear that public stakeholders have made a valuable contribution to it.

This is the first PEM research priority setting to use a validated framework such as the JLA, partnering with key stakeholders to design and lead the priority setting process from start to finish.

### Ethnic group sub-analysis

All questions selected for the workshop were reviewed to look for potential bias based on respondent demographic data. Question 8, discussing care for those with darker skin tones, was ranked highly for healthcare professionals but less so for members of the public. The steering group noted that the highest proportion of Survey two responses were from those identifying as white, 464 responses overall, with lower representation for people with lived experience from other ethnic groups (9%, 13/144). There was greater representation of other ethnic groups within the healthcare professional respondents which likely led to this discrepancy.

As such, a separate analysis was performed for participants who identified with any ethnic group other than white. The results of this sub-analysis showed question 8 did have a higher ranking in fifth position and had been under-represented with the initial analysis. Two other questions, question 13 about up-to-date technology and question 20 about ultrasound use, were also ranked higher in this sub-analysis but were not felt by the steering group to be unfairly biased by demographic representation.

These important considerations were brought to the workshop participants, which again had a larger proportion of individuals identifying as white.

### Strengths

This study has many strengths, including the use of the validated JLA methodology to partner with key stakeholders from start to finish of the research priority setting.

In particular, other PEM PSPs have had challenges with getting children and young person involvement. This is the first PEM PSP globally to link with an enthusiastic YPAG, to hear the voices of young people throughout the study. We were also able to successfully encourage participation from a wide range of children aged eight to 18 years old across both online surveys.

Another strength was the use of the shortlisting of 10 questions within Survey 2, which allows for greater specificity in the prioritisation process compared with Likert scales, which often results in closer grouping of all questions.

### Limitations

A limitation with this methodology is that the merging process and summarisation of questions can lead to broader questions which may lack the original question’s specificity. This can pose a challenge for the evidence review process, as broader questions will be less likely to have been answered. It also poses a potential problem later when researchers use the questions to apply for grant funding, as the relevant study designs may seem slightly detached from the broader question.

Other limitations for the survey design include the inability to detect duplicate responses for either survey if respondents remained anonymous, the inability to limit answers in Survey 2 and inability to complete the survey anonymously and sign-up to be informed of future participation opportunities. This may have discouraged respondents from taking part in further aspects of the study as they wished to remain anonymous.

## Conclusion

The PEM PSP has successfully achieved its aim of refreshing the Top 10 list of research priorities for PEM across the UK and Ireland (see table 3), building on the work from the first PERUKI prioritisation exercise but with a more rigorous methodological approach that included all key stakeholders. The final priority list reflects the views of healthcare professionals, patients and carers, with balanced priorities focusing on specific clinical topics and broader questions on delivery of care.

## Supplementary material

10.1136/emermed-2025-215836 online supplemental file 1

## Data Availability

Data are available upon reasonable request.
